# Correction: Global TALES feasibility study: Personal narratives in 10-year-old children around the world

**DOI:** 10.1371/journal.pone.0293705

**Published:** 2023-10-26

**Authors:** Marleen F. Westerveld, Rena Lyons, Nickola Wolf Nelson, Kai Mei Chen, Mary Claessen, Sara Ferman, Fernanda Dreux M. Fernandes, Gail T. Gillon, Khaloob Kawar, Jelena Kuvač Kraljević, Kakia Petinou, Eleni Theodorou, Tatiana Tumanova, Ioannis Vogandroukas, Carol Westby, on behalf the Global TALES Consortium

The 14th author’s name is spelled incorrectly. The correct name is: Ioannis Vogindroukas.
