# Supplementary material for: Global TALES feasibility study: Personal narratives in 10-year-old children around the world
Source: PLoS One. 2022 Aug 15;17(8):e0273114. doi: 10.1371/journal.pone.0273114 (PMC9377602; doi:10.1371/journal.pone.0273114)
Supplement: S1 Appendix — (DOCX) [file pone.0273114.s001.docx]

**Supplementary Appendix: Transcription reliability by country.**

| **Country** | **Process used for reliability checking** | **Utterance segmentation** | **Transcription Accuracy (words)** |
| --- | --- | --- | --- |
| Australia | All samples were first checked by a research assistant. An independent research assistant listened to 20% of the samples and checked for utterance segmentation and transcription accuracy | 95%  585/618 | 99%  6041/6105 |
| Brazil | Samples were transcribed by researcher and postgraduate student (phono-audiologist). Transcription and segmentation accuracy were reviewed – all disagreements were resolved through consensus. | Consensus | Consensus |
| Croatia | A postdoctoral student randomly selected 50% of the audiofiles and checked 100% of the written transcripts. | 97% | 99% |
| Cyprus | All transcripts were checked by two researchers for transcription accuracy and utterance segmentation. | > 85% | > 85% |
| Greece | The team discussed two samples in detail. Several samples were then exchanged between researchers and independently reviewed. | 88.7%  286/319 | 97.8%  2221/2271 |
| Israel_Arabic | Another examiner listened to all sound files – questions 1 and 6 only. | 100% 282/282 CUs | 98.8%  1370/1386 words |
| Israel_Hebrew | One examiner transcribed the responses to the problem narratives. 20% were checked by the main researcher.  Two examiners transcribed the remaining responses. 15% were checked by the main researcher. | No inaccuracies observed | No inaccuracies observed |
| New Zealand | All samples were first checked by a research assistant. An independent research assistant listened to 20% of the samples and checked for utterance segmentation and transcription accuracy | 99%  201/203 | 99%  1685/1696 |
| Russia | The samples were first checked by a research assistant. After that, 50% were checked and recalculated by the main researcher. | >87% | 98% |
| Taiwan | The main researcher transcribed all the samples. A graduate student checked 20% of the samples for transcription accuracy and utterance segmentation. | 96% | 98% |
| USA | All samples were first checked by a research assistant. An independent research assistant listened to 20% of the samples and checked for utterance segmentation and transcription accuracy | 96%  188/196 | 98%  1449/1471 |
