# Supplementary material for: Global TALES feasibility study: Personal narratives in 10-year-old children around the world
Source: PLoS One. 2022 Aug 15;17(8):e0273114. doi: 10.1371/journal.pone.0273114 (PMC9377602; doi:10.1371/journal.pone.0273114)
Supplement: S2 Appendix — (DOCX) [file pone.0273114.s002.docx]

**S2 Appendix: Topics assigned to the children’s responses by question (Phase 1)**

| **Topic:** | | **Description:** | **Example/s:** | |
| --- | --- | --- | --- | --- |
| **Question 1 - Excitement** | | | | |
|  | Family trip/holiday | the main topic is ‘going’ somewhere with family/family member | - going overseas | - going to a theme park |
|  | Personal achievement | achieved something that was specifically relevant to themselves | - being brave |  |
|  | Academic achievement | achieved something that was specifically relevant to school/grades | - good marks on a test | - completing an assignment |
|  | Sporting achievement | achieved something that was specifically relevant to sports | - winning a game | - working as a team |
|  | Moving/Relocating |  | - moving houses | - moving schools |
|  | Receiving new item/s | being given something new | - birthday present - buying new item | - new pet |
|  | Family event | anything specifically family related that marks a special day | - family birthday | - family gathering/reunion |
|  | Building/making new relationships | mentions of making new connections/relationships | - meeting a new friend | - feeling closer to someone |
|  | Spending time with friends/family | excitement stems from spending time with someone specific | - family | - friends |
|  | Learning new skill |  | - leaning a new game |  |
|  | Increased social status | being recognised in a more important way | - transition of boy of man |  |
|  | First experience | doing something for the first time | - first time playing ukulele |  |
|  | Reaching a personal goal | achievement a set goal | - scoring 3 goals in a game |  |
|  | Becoming independent | needing less support/help, given more responsibility | - walking home alone |  |
|  | Helping someone in need |  | - helping find something | - helping their nana clean |
|  | Reunion | seeing someone they haven’t see for a long time | - seeing and old friend |  |
|  | Friend’s birthday | specific to the birthday |  |  |
|  | Sibling achievement | when a sibling did well | - succeeding in production |  |

| **Question 2 - Worried** | | | | | |
| --- | --- | --- | --- | --- | --- |
|  | School based task | worries stemmed from something happening in school | - an upcoming test | - forgetting about homework | |
|  | Losing something/someone | not being able to find someone or an item | - losing mum shopping | - misplacing a toy | |
|  | Moving/relocating |  | - moving houses | - moving schools | |
|  | Conflicts/arguments | worries when hearing/seeing someone fighting | - parents fighting | - peers fighting | |
|  | Disappointing someone |  | - disappointing mum/dad by not achieving expectation | | |
|  | Not understanding new tasks | can be related to school, but not at school (see school-based task) | - not understanding homework | | |
|  | Injury/illness/medical condition | when they hurt themselves or talks about a medical condition | - broken arm their arm | - going to hospital again | |
|  | Choosing a side | needing to side in an argument/discussion |  |  | |
|  | Unexpected/new situation | unsure of what to do in a situation | - new sibling |  | |
|  | Uncertain of expectations |  | - given a new responsibility |  | |
|  | Causing damage to something |  | - breaking something | - marking something | |
|  | Honesty | owning up to something/taking responsibility | - telling parent they did something | | |
|  | Cancelled plans |  | - no longer going away | | - can’t see friend anymore |
|  | Family illness |  | - grandparent sick | |  |
|  | Family safety |  | - mum getting hurt doing something | | |
|  | Personal safety |  | - getting hurt walking home alone | | |
|  | Performance nerves | worried about having to perform in front of people | - first talent show | |  |
|  | Skill level | worried they won’t be able to | - not selected for a team | |  |
|  | Compromise | needing to do something they don’t want to for a friend/family | - changing the day the get to see their grandparents | | |
|  | being stolen from |  | - having a toy stolen from them | | |
|  | Pet illness/passing away |  | - needing to take pet to vet and not sure what will happen | | |
|  | Losing a friend |  |  | |  |
| **Question 3 - Annoyed** | | | | | |
|  | Differencing of opinions | where two parties are not agreeing | - can’t decide what to eat | |  |
|  | Siblings | story direct at annoying sibling or being annoyed by siblings | - When sister takes her things | |  |
|  | Skill level needed |  |  | |  |
|  | Not understanding | not being able to figure something out | - can’t understand instructions | | |
|  | Damage caused to something |  | - broke a toy | |  |
|  | Losing something/someone | not being able to find someone or an item | - losing mum shopping | | - misplacing a toy |
|  | Bullying |  | - being bullied themselves | | - seeing others bullied |
|  | Change of plans | plans cancelled or changed without their input | - not going to friends house anymore | | |
|  | Invasion of personal space | not being left alone | - sibling always in their room | |  |
|  | Academic responsibility | being expected to get certain results |  | |  |
|  | Permission refusal | being told no when asking to do something/get something | - wanting to go to a friend’s house but being told no | | |
|  | Copying of work | some copying their work | - having homework copied | |  |
|  | Differing expectations | expectations are different | - between siblings | | - between friends |
|  | Being stolen from |  |  | |  |
|  | Being overlooked | being ignored/overlooked by family/peers | - being ignored at school by friends | | |
|  | Conflict between friends/family |  | - friends/family fighting | |  |
|  | Peer relationship | stories relevant to interactions and reactions of peer relationships | - helping a friend | | - trying to fix a friends fight |
|  | Jealousy |  | - wanting something someone else has | | |
|  | Illness/injury/medical condition | when they hurt themselves or talks about a medical condition | - broken arm so can’t play sports | | |
|  | Being lied to |  | - lied to about what a friend said about them | | |

| **Question 4 - Proud** | | | | |
| --- | --- | --- | --- | --- |
|  | Academic achievement | achieved something that was specifically relevant to school/grades | - good marks on a test | - completing an assignment |
|  | Personal achievement | achieved something that was specifically relevant to themselves | - being brave |  |
|  | Sporting achievement | achieved something that was specifically relevant to sports | - winning a game | - working as a team |
|  | Musical achievement | achieved something the was specifically relevant to music | - learning new song on the guitar | |
|  | Helping someone/something in need |  | - helping find something - standing up for friend | - helping their nana clean |
|  | Receiving praise | achievements being verbally acknowledged |  |  |
|  | Sibling being born |  |  |  |
|  | Personal initiative | doing something helpful without being asked | - cleaning the house before mum gets home | |
|  | Overcoming fear/conflict |  | - resolving a fight | - talking in front of the class |
|  | Honesty | owning up to something/taking responsibility | - telling parent they did something | |
|  | Learning new skill |  | - learning a new game |  |
|  | Personal growth |  | - getting a role in a play - learning to knit | |

| **Question 5 – Problem** | | | | |
| --- | --- | --- | --- | --- |
|  | Peer relationship | stories relevant to interactions and reactions of peer relationships | - not talking to a classmate | - fighting with friends |
|  | Fixing/making/replacing something |  | - fixing something they broke | |
|  | Differences of opinion | where two parties are not agreeing | - can’t decide what to eat, but work it out | |
|  | Illness/injury/medical condition | overcoming an illness/injury/medical condition |  |  |
|  | School based task | problem stemmed from something happening in school | - an upcoming test | - forgetting homework |
|  | Changing of plans | needing to cope with a change of plans | - change/adapt plan for something that’s changed | |
|  | Being copied |  | - classmate copying work |  |
|  | Breaking a rule | consequences/resolving breaking a room | - apologising for doing something wrong | |
|  | Making a mistake |  | - apologising for doing something wrong | |
|  | Family/friend support/involvement | needing more support/amount of support from friends/family | - needing specific help from mum | |
|  | Bullying | resolving/confronting bullying | - standing up for a bullied friend | |
|  | Personal wellness |  | - keeping getting sick | - picking a safer way home |
|  | Emotional issues | unable to control emotions/done know how to respond | - trying to stop crying a lot |  |
|  | Loosing something/someone | working to resolve not being able to find someone or an item | - working to find someone |  |
|  | Changing tactic / mentality | having to change way of thinking to complete something | - learning new rule/skill |  |
|  | Honesty | owning up to something/taking responsibility | - telling parent they did something | |

| **Question 6 - Important** | | | | |
| --- | --- | --- | --- | --- |
|  | Social achievement |  |  |  |
|  | sporting achievement | achieved something that was specifically relevant to sports | - winning a game | - working as a team |
|  | Family event | anything specifically family related that marks a special day | - family birthday | - family gathering/reunion |
|  | Family/friend support/involvement | support/amount of support from friends/family | - cheering them on in a game | |
|  | personal achievement | achieved something that was specifically relevant to themselves | - being brave |  |
|  | cultural involvement | participating in cultural activities | - doing kapa haka (NZ) |  |
|  | academic achievement | achieved something that was specifically relevant to school/grades | - good marks on a test | - completing an assignment |
|  | expression of love/thanks |  | - parent saying how much they love them | |
|  | saving someone/something |  | - calling an ambulance |  |
|  | overcoming fear | doing something that scares them |  |  |
|  | building/making new relationships | mentions of making new connections/relationships | - meeting a new friend | - feeling closer to someone |
|  | receiving new item | being given something new | - birthday present | - new pet |
|  | family holiday/trip | the main topic is ‘going’ somewhere with family/family member | - going overseas | - going to a theme park |
|  | peer relationships | important interactions and reactions of their peers | - not talking to a classmate | - fighting with friends |
|  | reconciliation | fixing relationships | - becoming friends again |  |
|  | musical achievement | achieved something the was specifically relevant to music | - learning new song on the guitar | |
|  | personal wellness | overcoming a sickness/injury | - keeping getting sick | - picking a safer way home |
