# Supplementary material for: Global TALES feasibility study: Personal narratives in 10-year-old children around the world
Source: PLoS One. 2022 Aug 15;17(8):e0273114. doi: 10.1371/journal.pone.0273114 (PMC9377602; doi:10.1371/journal.pone.0273114)
Supplement: S3 Appendix — (DOCX) [file pone.0273114.s003.docx]

**S3 Appendix: Consolidated criteria for reporting qualitative studies (COREQ): 32-item checklist (Tong et al., 2007)**

**Domain 1: Research team and reflexivity**

|  | **Number and item** | **Guide questions/description** | **Response** |
| --- | --- | --- | --- |
| Personal Characteristics | 1. Interviewer/facilitator | Which author/s conducted the interview or focus group? | NT (research assistant). |
|  | 2. Credentials | What were the researcher’s credentials? E.g. PhD, MD | Bachelor of Speech and Language Therapy with Honours - BSLP(Hons). |
|  | 3. Occupation | What was their occupation at the time of the study? | Speech therapist and researcher. |
|  | 4. Gender | Was the researcher male or female? | Female. |
|  | 5. Experience and training | What experience or training did the researcher have? | Experience with using the Global TALES protocol with students in NZ aged from 10-13 years. |
| Relationship with participants | 6. Relationship established | Was a relationship established prior to study commencement? | One of the participants was known to the researcher prior to the interviews. The interview guide started with introductions. |
|  | 7. Participant knowledge of the interviewer | What did the participants know about the researcher? E.g. personal goals, reasons for doing the research | Participants were provided with a Participant Information Leaflet prior to participating in the study, which included the rationale for the research. They were also given the key interview guide questions in advance of the interview. |
|  | 8. Interviewer characteristics | What characteristics were reported about the interviewer/facilitator? e.g. Bias, assumptions, reasons and interests in the research topic | Three members of the research team (MW, RL and NT) worked together to design the interview guide. The team held positive views about the project and were careful to ensure that the interview guide contained open-ended questions where participants could discuss both positive and negative experiences. |

**Domain 2: Study design**

| Theoretical framework | 9. Methodological orientation and Theory | What methodological orientation was stated to underpin the study? e.g. grounded theory, discourse analysis, ethnography, phenomenology, content analysis. | Content analysis was used. |
| --- | --- | --- | --- |
| Participant selection | 10. Sampling | How were participants selected? e.g. purposive, convenience, consecutive, snowball | Purposive sampling with the following inclusion criteria: participants who had plans to collect and analyse personal narratives from a sample of 10 year children in their respective countries; and participants who had already collected and analysed these personal narratives. |
|  | 11. Method of approach | How were participants approached? e.g. face-to-face, telephone, mail, email | Email. |
|  | 12. Sample size | How many participants were in the study? | Participants from 12 countries, with 18 participants in total. |
|  | 13. Non-participation | How many people refused to participate or dropped out? Reasons? | One interviewee was unable to participate. |
| Setting | 14. Setting of data collection | Where was the data collected? e.g., home, clinic, workplace | Online at an agreed time (negotiated to accommodate time differences). |
|  | 15. Presence of non-participants | Was anyone else present besides the participants and researchers? | No. |
|  | 16. Description of sample | What are the important characteristics of the sample? e.g. demographic data, date | Participants from 12 countries, with 17 participants in total including the Lead Investigator in each country with speech pathologists, research assistants and/or students in 4 countries. All of the Lead Investigators held a qualification in speech and language therapy. |
| Data collection | 17. Interview guide | Were questions, prompts, guides provided by the authors? Was it pilot tested? | Some of the key questions were sent to the participants in advance of the interview. A pilot was not conducted. It was agreed that the interviews would be conducted in English. |
|  | 18. Repeat interviews | Were repeat interviews carried out? If yes, how many? | No. |
|  | 19. Audio/visual recording | Did the research use audio or visual recording to collect the data? | The interviews were conducted and audio-recorded on Zoom and using an audio recorder. Recordings were transcribed using the otter.ai programme and then manually double-checked. |
|  | 20. Field notes | Were field notes made during and/or after the interview or focus group? | No. |
|  | 21. Duration | What was the duration of the interviews or focus group? | Ranged from 14.30 to 32.11 minutes (mean: 24.75). |
|  | 22. Data saturation | Was data saturation discussed? | Not applicable for this study. |
|  | 23. Transcripts returned | Were transcripts returned to participants for comment and/or correction? | No. |

**Domain 3: Analysis and findings**

| Data analysis | 24. Number of data coders | How many data coders coded the data? | One (RL). |
| --- | --- | --- | --- |
|  | 25. Description of the coding tree | Did authors provide a description of the coding tree? | Yes. |
|  | 26. Derivation of themes | Were themes identified in advance or derived from the data? | Categories were derived from the data. |
|  | 27. Software | What software, if applicable, was used to manage the data? | NVIVO 12. |
|  | 28. Participant checking | Did participants provide feedback on the findings? | Yes, participants were requested to: review the final draft categories; and comment on whether or not the analysis represented their views. |
| Reporting | 29. Quotations presented | Were participant quotations presented to illustrate the themes / findings? Was each quotation identified? e.g., participant number | Yes. |
|  | 30. Data and findings consistent | Was there consistency between the data presented and the findings? | Yes, and this was confirmed by the participants. |
|  | 31. Clarity of major themes | Were major themes clearly presented in the findings? | Categories were explained and supported with data. |
|  | 32. Clarity of minor themes | Is there a description of diverse cases or discussion of minor themes? | Not applicable. |
