# Supplementary material for: Global TALES feasibility study: Personal narratives in 10-year-old children around the world
Source: PLoS One. 2022 Aug 15;17(8):e0273114. doi: 10.1371/journal.pone.0273114 (PMC9377602; doi:10.1371/journal.pone.0273114)
Supplement: S4 Appendix — (DOCX) [file pone.0273114.s004.docx]

**S4 Appendix: Global TALES interview guide.**

| **Topic** | **Questions** |
| --- | --- |
| Experiences of translating the protocol and views on its cultural appropriateness in the participating countries | - When you were given the English protocol, how did you go about translating it into your language? - Can you give any examples about the challenges you faced?   - Were there some words in particular that were hard to transcribe? If so, what were they?   - Were there any topics/prompts that were not applicable to your country/language? Please explain. - Can you give any examples of where it went really well or where it was really straight forward? - Do you feel the translated protocol reflected the content of the English one? Did you make any changes? Please provide examples. - Did the protocol fit culturally once translated? Were they appropriate questions for children in your country? Can you tell me more about that? Can you give me an example(s)? |
| Experiences of using the protocol to elicit narratives | - Did you do it yourself or get someone else to do it? If someone else helped, who? - What prompts do you feel worked well? Which didn’t? Please give example(s). - Were there questions that kids did not want to talk about, etc.? - Would you have added other prompts? Were any not needed? |
| Experiences of the transcription and analytical processes | - What process do you use for transcription? - How do you typically analyse samples in your language? (computerised, by hand, MS Word …). - What information would you like to get out of the analysis personally? Explain please |
| Views on next steps for the Global TALES project | - Do you feel the Global TALES protocol might be useful in your country? How? Why not? - What further development would you like to see? - Looking back, was there anything else that would have been helpful to support you further with the process of translating and using the Global TALES protocol in your language/culture? |
