# Supplementary material for: Global TALES feasibility study: Personal narratives in 10-year-old children around the world
Source: PLoS One. 2022 Aug 15;17(8):e0273114. doi: 10.1371/journal.pone.0273114 (PMC9377602; doi:10.1371/journal.pone.0273114)
Supplement: S5 Appendix — (DOCX) [file pone.0273114.s005.docx]

**S5 Appendix. Global TALES Protocol v2**

**Elicitation Protocol**

*I am going to show you six prompts.* (Prompts are typed on laminated cards or presented on an iPad/Tablet and presented in a set order). *I will read each prompt out loud. Each prompt asks you to tell a story about something that has happened to you. I want you to tell me as much about it as you can, so that I can get a very clear idea of what was happening, but it doesn’t have to be really big or extra special.*

*Listen to each prompt and think about a time that you can tell me about. When you are ready to start, let me know and I will turn on the recorder. You can take as long as you like.*

Read the initial prompt; then wait for approximately 5 seconds. Don’t wait too long – no more than 10 secs.

If the child is struggling to think of something (i.e., there is a pause of more than 5 seconds, or the child says, “*I don’t know,”* then add the scripted follow-up prompt that is provided for each story prompt.

If the child responds to the initial prompt with a topic (e.g., “*yes, I won a prize,*” or “*my brother is annoying sometimes,*” then follow up with the general prompt:

*Tell me a story about that!*

After using this general prompt, try just showing an interested and encouraging face. Repeat the general prompt if nothing is forthcoming, or follow-up with the following additional prompts if the child only provides 1 or 2 sentences:

*Can you tell me more?*

*I would like to know more about your story. Is there anything else you can tell me?*

*Can you explain what you mean by that?*

You can also use generic and back-channel encouragements to encourage the child to continue talking. Make sure the prompts are neutral (not leading). For example, you may say things like:

*Wow*

*That’s interesting!*

*Uh-huh*

You may repeat something the child just told you (without adding new information). For example *“your test was really hard”.*

*“you were preparing for a race”.*

Do NOT ask leading questions and avoid giving specific prompts, such as “*How does your story end?*”

Make sure you ask all six protocol prompts using the set order (1 to 6).

**Protocol Prompts and scripted follow-up prompts:**

1. **Tell me a story about a time when you felt excited, or really happy.**

Scripted follow-up prompt (if needed) - Other children tell me about a special celebration, like a family party, or sometimes they tell me about a special holiday or family vacation they had.

1. **Tell me a story about a time when you felt worried or confused. Perhaps a time when lots of things were happening and you didn’t know what to do.**

Scripted follow-up prompt (if needed) - Other children tell me about times when they have had to do a project for school, or sometimes they tell me about when they have moved house.

1. **Tell me a story about a time when you were really annoyed or angry.**

Scripted follow-up prompt (if needed) - Other children tell me a story about a friend or a brother or sister. Or sometimes they tell me about someone from the classroom who really annoyed them or made them feel angry.

1. **Tell me a story about a time when you felt proud of yourself.**

Scripted follow-up prompt (if needed) - Some children tell me about at time they were kind to someone else, or about a time they worked hard and won a prize.

1. **Tell me a story about a time when you had a problem and you had to fix it. Tell me all about what happened and what you had to do to fix it.**

Scripted follow-up prompt (if needed) - Other children tell me about a time when they helped somebody who had a problem, or when they faced a problem and had to figure out what to do about it.

1. **Tell me a story about something that has happened to you that was very important to you.**

Scripted follow-up prompt (if needed) - Some children tell me about winning something, or maybe a time when they did very well at school.
