# Supplementary material for: Global TALES feasibility study: Personal narratives in 10-year-old children around the world
Source: PLoS One. 2022 Aug 15;17(8):e0273114. doi: 10.1371/journal.pone.0273114 (PMC9377602; doi:10.1371/journal.pone.0273114)
Supplement: S1 Table — (DOCX) [file pone.0273114.s006.docx]

|  | | | | | | | | | | | | | | | | | | | | | | | | | | | | | | | | | | |  |  |
| --- | --- | --- | --- | --- | --- | --- | --- | --- | --- | --- | --- | --- | --- | --- | --- | --- | --- | --- | --- | --- | --- | --- | --- | --- | --- | --- | --- | --- | --- | --- | --- | --- | --- | --- | --- | --- |
| **S1 Table. Productivity measures by country by protocol prompt** | | | | | | | | | | | | | | | | | | | | | | | | | | | | | | | | | | |  |  |
|  | |  |  |  |  | | | **Total number of Utts** | | **TNW Totals** | | **Utts**  **P1** | | **TNW P1** | | **Utts P2** | | **TNW P2** | | **Utts**  **P3** | | **TNW**  **P3** | | **Utts P4** | | **TNW P4** | | **Utts**  **P5** | | **TNW**  **P5** | | **Utts**  **P6** | | **TNW**  **P6** | | |
|  | **Australia** | | | | | Mean | 68.75 | | 622.45 | | 13.30 | | 114.75 | | 11.38 | | 105.93 | | 10.65 | | 95.85 | | 10.10 | | 93.58 | | 13.28 | | 121.08 | | 10.05 | | 91.28 | | |  |
|  |  |  |  |  |  | SD | 27.49 | | 267.84 | | 8.06 | | 78.37 | | 5.63 | | 52.23 | | 5.20 | | 48.61 | | 5.41 | | 49.35 | | 7.23 | | 70.16 | | 6.71 | | 58.34 | | |  |
|  |  |  |  |  |  | Min | 25.00 | | 223.00 | | 2.00 | | 18.00 | | 4.00 | | 31.00 | | 2.00 | | 22.00 | | 2.00 | | 24.00 | | 1.00 | | 6.00 | | .00 | | .00 | | |  |
|  |  |  |  |  |  | Max | 123.00 | | 1251.0 | | 39.00 | | 377.00 | | 24.00 | | 218.00 | | 26.00 | | 241.00 | | 24.00 | | 248.00 | | 32.00 | | 280.00 | | 33.00 | | 295.00 | | |  |
|  | **Brazil** | | | | | Mean | 51.10 | | 427.29 | | 8.43 | | 70.81 | | 9.38 | | 82.57 | | 9.29 | | 61.00 | | 5.86 | | 50.90 | | 8.90 | | 71.76 | | 9.24 | | 90.24 | | |  |
|  |  |  |  |  |  | SD | 37.63 | | 384.90 | | 6.56 | | 57.05 | | 7.05 | | 99.06 | | 6.61 | | 46.64 | | 5.53 | | 52.42 | | 5.28 | | 44.83 | | 10.38 | | 145.33 | | |  |
|  |  |  |  |  |  | Min | 22.00 | | 166.00 | | 3.00 | | 17.00 | | 2.00 | | 18.00 | | 3.00 | | 17.00 | | 2.00 | | 10.00 | | 4.00 | | 30.00 | | 1.00 | | 10.00 | | |  |
|  |  |  |  |  |  | Max | 199.00 | | 1918.0 | | 33.00 | | 284.00 | | 33.00 | | 446.00 | | 29.00 | | 216.00 | | 28.00 | | 224.00 | | 26.00 | | 224.00 | | 50.00 | | 697.00 | | |  |
|  | **Croatia** | | | | | Mean | 45.81 | | 353.48 | | 7.81 | | 59.70 | | 7.96 | | 62.33 | | 7.22 | | 56.44 | | 7.96 | | 60.11 | | 8.07 | | 59.56 | | 6.74 | | 55.33 | | |  |
|  |  |  |  |  |  | SD | 27.31 | | 194.42 | | 5.01 | | 31.62 | | 7.48 | | 55.68 | | 3.77 | | 33.93 | | 8.53 | | 58.01 | | 4.21 | | 30.18 | | 3.25 | | 27.02 | | |  |
|  |  |  |  |  |  | Min | 25.00 | | 166.00 | | 3.00 | | 19.00 | | 2.00 | | 22.00 | | 2.00 | | 21.00 | | 1.00 | | 8.00 | | 2.00 | | 7.00 | | 1.00 | | 16.00 | | |  |
|  |  |  |  |  |  | Max | 169.00 | | 1217.0 | | 27.00 | | 181.00 | | 42.00 | | 317.00 | | 20.00 | | 174.00 | | 47.00 | | 315.00 | | 17.00 | | 138.00 | | 16.00 | | 136.00 | | |  |
|  | **Cyprus** | | | | | Mean | 80.79 | | 396.68 | | 11.89 | | 52.32 | | 10.42 | | 48.89 | | 16.21 | | 81.95 | | 12.26 | | 58.21 | | 16.05 | | 85.89 | | 13.95 | | 71.74 | | |  |
|  |  |  |  |  |  | SD | 47.06 | | 251.71 | | 5.92 | | 27.76 | | 5.72 | | 26.90 | | 13.57 | | 73.23 | | 12.00 | | 52.08 | | 14.13 | | 81.90 | | 10.23 | | 54.83 | | |  |
|  |  |  |  |  |  | Min | 23.00 | | 106.00 | | 4.00 | | 16.00 | | 2.00 | | 6.00 | | 2.00 | | 13.00 | | .00 | | .00 | | .00 | | .00 | | .00 | | .00 | | |  |
|  |  |  |  |  |  | Max | 201.00 | | 1123.0 | | 22.00 | | 104.00 | | 21.00 | | 98.00 | | 64.00 | | 326.00 | | 54.00 | | 227.00 | | 59.00 | | 345.00 | | 37.00 | | 183.00 | | |  |
|  | **Greece** | | | | | Mean | 63.80 | | 428.35 | | 9.45 | | 63.00 | | 11.15 | | 76.95 | | 10.30 | | 68.30 | | 8.55 | | 59.55 | | 12.60 | | 89.65 | | 11.75 | | 70.90 | | |  |
|  |  |  |  |  |  | SD | 22.91 | | 171.35 | | 4.07 | | 32.38 | | 5.47 | | 36.54 | | 6.94 | | 44.94 | | 4.48 | | 35.01 | | 6.61 | | 47.43 | | 4.78 | | 33.46 | | |  |
|  |  |  |  |  |  | Min | 31.00 | | 185.00 | | 4.00 | | 17.00 | | 3.00 | | 20.00 | | 3.00 | | 22.00 | | 4.00 | | 20.00 | | 4.00 | | 31.00 | | 3.00 | | 26.00 | | |  |
|  |  |  |  |  |  | Max | 126.00 | | 842.00 | | 22.00 | | 152.00 | | 25.00 | | 152.00 | | 34.00 | | 234.00 | | 18.00 | | 130.00 | | 30.00 | | 202.00 | | 24.00 | | 133.00 | | |  |
|  | **Israel_Arabic** | | | | | Mean | 43.70 | | 201.90 | | 6.20 | | 28.55 | | 8.20 | | 37.00 | | 7.20 | | 31.70 | | 6.05 | | 30.55 | | 7.35 | | 33.55 | | 7.90 | | 40.05 | | |  |
|  |  |  |  |  |  | SD | 10.61 | | 56.66 | | 3.52 | | 14.26 | | 3.30 | | 14.12 | | 3.58 | | 19.10 | | 3.63 | | 16.08 | | 3.50 | | 16.02 | | 2.99 | | 18.56 | | |  |
|  |  |  |  |  |  | Min | 23.00 | | 108.00 | | 2.00 | | 8.00 | | 3.00 | | 15.00 | | 3.00 | | 9.00 | | 2.00 | | 8.00 | | 2.00 | | 9.00 | | 4.00 | | 17.00 | | |  |
|  |  |  |  |  |  | Max | 71.00 | | 316.00 | | 19.00 | | 75.00 | | 15.00 | | 63.00 | | 19.00 | | 94.00 | | 17.00 | | 64.00 | | 19.00 | | 74.00 | | 14.00 | | 94.00 | | |  |
|  | **Israel_Hebrew** | | | | | Mean | 50.40 | | 385.45 | | 8.75 | | 67.65 | | 8.80 | | 65.60 | | 6.90 | | 52.55 | | 8.55 | | 69.10 | | 9.15 | | 74.20 | | 8.00 | | 56.35 | | |  |
|  |  | | | | | SD | 12.86 | | 150.52 | | 5.46 | | 44.80 | | 4.81 | | 40.57 | | 2.65 | | 30.42 | | 4.50 | | 47.95 | | 3.99 | | 45.90 | | 3.28 | | 24.28 | | |  |
|  |  | | | | | Min | 30.00 | | 174.00 | | 3.00 | | 14.00 | | .00 | | .00 | | .00 | | .00 | | 4.00 | | 21.00 | | 4.00 | | 25.00 | | 4.00 | | 24.00 | | |  |
|  |  | | | | | Max | 80.00 | | 668.00 | | 22.00 | | 171.00 | | 18.00 | | 145.00 | | 12.00 | | 117.00 | | 23.00 | | 221.00 | | 19.00 | | 220.00 | | 17.00 | | 117.00 | | |  |
|  | **New Zealand** | | | | | Mean | 55.75 | | 182.00 | | 9.35 | | 79.20 | | 12.45 | | 105.15 | | 9.70 | | 81.75 | | 9.40 | | 75.80 | | 8.65 | | 71.75 | | 8.60 | | 70.15 | | |  |
|  |  |  |  |  |  | SD | 20.57 | | 43.20 | | 4.43 | | 32.08 | | 20.02 | | 159.41 | | 5.04 | | 36.78 | | 6.13 | | 46.85 | | 5.84 | | 47.94 | | 5.67 | | 45.02 | | |  |
|  |  |  |  |  |  | Min | 35.00 | | 124.00 | | 4.00 | | 42.00 | | 2.00 | | 13.00 | | 5.00 | | 41.00 | | 3.00 | | 24.00 | | .00 | | .00 | | .00 | | .00 | | |  |
|  |  |  |  |  |  | Max | 114.00 | | 277.00 | | 21.00 | | 177.00 | | 96.00 | | 769.00 | | 24.00 | | 164.00 | | 27.00 | | 202.00 | | 27.00 | | 221.00 | | 21.00 | | 143.00 | | |  |
|  | **Russia** | | | | | Mean | 62.30 | | 475.60 | | 10.25. | | 83.60 | | 9.20 | | 79.75. | | 10.35. | | 82.20. | | 13.45 | | 78.05 | | 10.25 | | 88.50 | | 8.80 | | 63.50 | | |  |
|  |  |  |  |  |  | SD | 32.78 | | 261.55 | | 6.34. | | 62.51. | | 6.43. | | 57.25. | | 5.35. | | 48.63. | | 16.91 | | 50.60 | | 5.85 | | 48.92 | | 6.97 | | 46.46 | | |  |
|  |  |  |  |  |  | Min | 17.00 | | 118.00 | | 3.00. | | 12.00. | | 2.00. | | 14.00. | | 3.00. | | 15.00. | | 2.00 | | 12.00 | | 1.00 | | 7.00 | | 3.00 | | 16.00 | | |  |
|  |  |  |  |  |  | Max | 131.00 | | 1137.0 | | 27.00. | | 220.0. | | 23.00. | | 196.0. | | 21.00. | | 180.0. | | 80.00 | | 186.0 | | 22.00 | | 198.00 | | 23.00 | | 201.0 | | |  |
|  | **Taiwan** | | | | | Mean | 61.00 | | 518.10 | | 12.10 | | 104.60 | | 11.30 | | 95.85 | | 10.65 | | 91.70 | | 7.80 | | 65.65 | | 10.45 | | 84.55 | | 8.70 | | 75.75 | | |  |
|  |  |  |  |  |  | SD | 45.59 | | 481.55 | | 14.15 | | 139.54 | | 11.54 | | 117.56 | | 11.23 | | 120.78 | | 5.12 | | 49.35 | | 6.59 | | 58.17 | | 4.81 | | 41.70 | | |  |
|  |  |  |  |  |  | Min | 22.00 | | 158.00 | | 2.00 | | 17.00 | | 2.00 | | 18.00 | | 3.00 | | 19.00 | | 3.00 | | 14.00 | | 3.00 | | 21.00 | | 3.00 | | 26.00 | | |  |
|  |  |  |  |  |  | Max | 227.00 | | 2348.0 | | 66.00 | | 624.00 | | 55.00 | | 565.00 | | 54.00 | | 573.00 | | 19.00 | | 201.00 | | 26.00 | | 234.00 | | 22.00 | | 158.00 | | |  |
|  | **USA** | | | | | Mean | 54.09 | | 160.09 | | 7.55 | | 66.91 | | 8.86 | | 73.86 | | 9.36 | | 78.86 | | 8.55 | | 69.18 | | 11.14 | | 97.86 | | 8.86 | | 72.55 | | |  |
|  |  |  |  |  |  | SD | 36.67 | | 68.45 | | 4.38 | | 46.19 | | 5.37 | | 53.62 | | 8.85 | | 95.42 | | 5.47 | | 59.22 | | 6.95 | | 70.23 | | 5.35 | | 46.07 | | |  |
|  |  |  |  |  |  | Min | 19.00 | | 71.00 | | .00 | | .00 | | 3.00 | | 26.00 | | 3.00 | | 23.00 | | 3.00 | | 12.00 | | 5.00 | | 18.00 | | .00 | | .00 | | |  |
|  |  |  |  |  |  | Max | 185.00 | | 365.00 | | 20.00 | | 179.00 | | 26.00 | | 262.00 | | 41.00 | | 463.00 | | 26.00 | | 286.00 | | 36.00 | | 357.00 | | 21.00 | | 208.00 | | |  |

P1 = protocol prompt 1; Utts = utterances; TNW = Total number of words
