# Supplementary figures and images for: Global TALES feasibility study: Personal narratives in 10-year-old children around the world
Source: PLoS One. 2022 Aug 15;17(8):e0273114. doi: 10.1371/journal.pone.0273114 (PMC9377602; doi:10.1371/journal.pone.0273114)

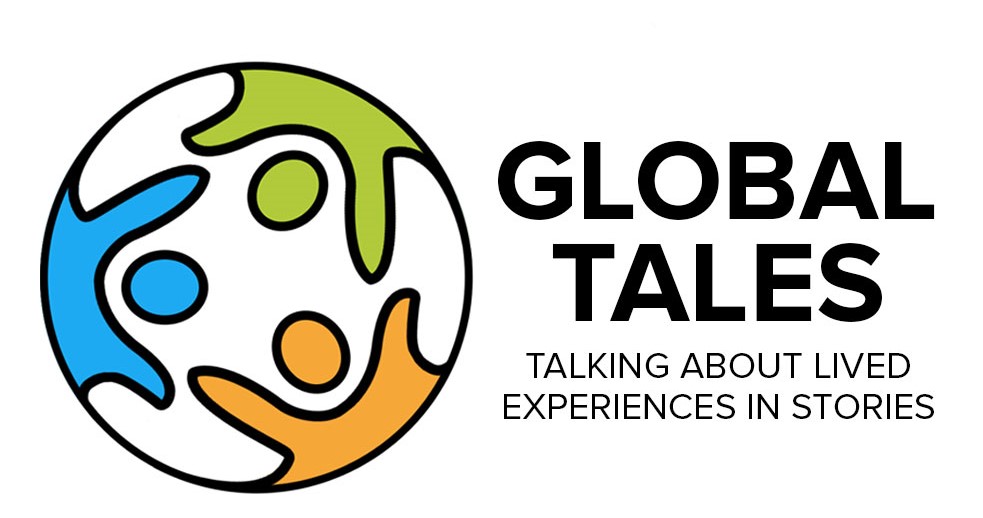

Supplement: S1 Fig — (JPG) [file pone.0273114.s007.jpg]
