# Supplementary material for: Global TALES feasibility study: Personal narratives in 10-year-old children around the world
Source: PLoS One. 2022 Aug 15;17(8):e0273114. doi: 10.1371/journal.pone.0273114 (PMC9377602; doi:10.1371/journal.pone.0273114)
Supplement: Attachment — Submitted filename: Rebuttal Letter PONE D 22 10735 final.docx [file pone.0273114.s009.docx]

**To the Editor**

Please find our detailed responses to the editor’s and the reviewers’ comments below. This accompanies our cover letter.

**Journal Requirements:**

When submitting your revision, we need you to address these additional requirements.

1. Please ensure that your manuscript meets PLOS ONE's style requirements, including those for file naming. The PLOS ONE style templates can be found at 
[https://journals.plos.org/plosone/s/file?id=wjVg/PLOSOne_formatting_sample_main_body.pdf](https://protect-au.mimecast.com/s/HXLiCvl0KYH7QoW13hAXRf2?domain=journals.plos.org) and

[https://journals.plos.org/plosone/s/file?id=ba62/PLOSOne_formatting_sample_title_authors_affiliations.pdf](https://protect-au.mimecast.com/s/BDutCwVLMEfGBXL6WI1DR1X?domain=journals.plos.org)

**Author response**:

- We have carefully checked all style requirements and sincerely hope we haven’t missed anything. Some guidelines were ambiguous such as the use of headings or paragraph indentations.
- Please note that some minor corrections have been made to author names and affiliations.

**2. Thank** you for stating the following in the Competing Interests section:

"MW has a financial relationship with SALT Software. The remaining authors have declared that no competing interests exist."

We note that you received funding from a commercial source: SALT Software

Please provide an amended Competing Interests Statement that explicitly states this commercial funder, along with any other relevant declarations relating to employment, consultancy, patents, products in development, marketed products, etc.

Within this Competing Interests Statement, please confirm that this does not alter your adherence to all PLOS ONE policies on sharing data and materials by including the following statement: "This does not alter our adherence to PLOS ONE policies on sharing data and materials.” (as detailed online in our guide for authors [http://journals.plos.org/plosone/s/competing-interests](https://protect-au.mimecast.com/s/RFS6CxnMOVU1YKJ83TWlC-H?domain=journals.plos.org)).  If there are restrictions on sharing of data and/or materials, please state these. Please note that we cannot proceed with consideration of your article until this information has been declared.

Please include your amended Competing Interests Statement within your cover letter. We will change the online submission form on your behalf.

**Author response**: I have amended the Statement and have included this in the cover letter as requested. Of note, we did not receive funding from SALT Software LLC.

**3. One of** the noted authors is a group or consortium "Global TALES Consortium". In addition to naming the author group, please list the individual authors and affiliations within this group in the acknowledgments section of your manuscript. Please also indicate clearly a lead author for this group along with a contact email address.

**Author Response**: All named authors are members of the Global TALES Consortium with the main authors named on the author page. Additional members have been named in the acknowledgements, along with their affiliations. There is no lead author for this *additional* group. We have made this clearer by adding ‘Additional’ to read: Additional members of the Global TALES Consortium. I (Marleen Westerveld am the corresponding author and the leader of the Global TALES Consortium).

**4. Please review** your reference list to ensure that it is complete and correct. If you have cited papers that have been retracted, please include the rationale for doing so in the manuscript text, or remove these references and replace them with relevant current references. Any changes to the reference list should be mentioned in the rebuttal letter that accompanies your revised manuscript. If you need to cite a retracted article, indicate the article’s retracted status in the References list and also include a citation and full reference for the retraction notice.

**Author response:** the reference list has been checked and all journal names have been changed to adhere to the PLOS ONE style guidelines. No other changes have been made, except for adding a reference in response to reviewer one’s comment (see below). No retracted articles were included.

**Editor Comments**
It was a pleasure reading your manuscript. Two expert reviewers and myself reviewed the paper and agree that it is going to be an important contribution to the literature and potentially clinical practice. I would like to provisionally accept your paper after some very minor revisions. Please address these issues and we will move forward.

We would like to see you include more information for the reader on how you envision the protocol being used in research and clinical practice across culturally and linguistically diverse populations. Along these lines, the reader would profit from more discussion of the pros and cons of greater standardization guidelines for consistent administration of the protocol. One minor edit that should be made is line 100; 3 year olds were not included in the study that you reference, please insert the proper reference. Also, please edit the manuscript so that Tales is stated as "Tales" or "TALES" but not both.

**Author response:** We have responded to these issues in our response to the individual reviewers below. In summary, we have re-arranged the final two pages of the manuscript and added two paragraphs to the final section of the paper.

As suggested, we have added a reference to support the inclusion of 3-year-olds. We have now consistently referred to the protocol using capital letters for TALES.

Thank you for your very positive feedback.


**Reviewer's Responses to Questions**

**3. Have the** authors made all data underlying the findings in their manuscript fully available?

The [PLOS Data policy](https://protect-au.mimecast.com/s/YgjqCyoNQ6CrxENDRUPG9tC?domain=plosone.org) requires authors to make all data underlying the findings described in their manuscript fully available without restriction, with rare exception (please refer to the Data Availability Statement in the manuscript PDF file). The data should be provided as part of the manuscript or its supporting information, or deposited to a public repository. For example, in addition to summary statistics, the data points behind means, medians and variance measures should be available. If there are restrictions on publicly sharing data—e.g. participant privacy or use of data from a third party—those must be specified.

Reviewer #1: Yes

Reviewer #2: Yes

**Author Response:** We have now uploaded an Excel file containing the data pertaining to Research Question 1 to our OSF site.

**Reviewer #1: PLOS ONE**

Minor points:

• Sometimes project is referred to as TALES, sometimes Tales; best to standardize.

**Author Response**: this has been corrected – now referred to as TALES throughout.

• Authors used the word “story” in elicitation, whereas “memory” might have been better, but it is hard to quibble with success.

**Author Response**: thank you for that suggestion. We agreed to using ‘story’ when developing the protocol so have not changed the wording at this stage of our project.

• Other edits are in appended pdf document.

**Response:** thank you. All minor edits have been responded to in the R1 of the paper. See the track changes version of the paper.

**Reviewer #2:** 
I would like to see a bit more about how the authors envision the uses of the protocol. The analyses in the current paper focused on word and C-unit counts, and that was appropriate for this first pass. It could conceivably be used to compare content of personal stories in terms of cultural content and so forth. The authors allude to clinical uses only indirectly, by mention of making the tool “more attractive for clinicians”.

**Author response:**

The conclusion sections have been restructured. We have also added the following paragraph to make this clearer:

These findings will also be important for clinicians who work with children from culturally and/or linguistically diverse backgrounds on a daily basis. Eventually, country-specific data, reflecting differences within and across countries and cultures and evaluated for reliability and validity, could result in a practical tool for timely identification of developmental language disorder. From a clinical perspective, having access to an ecologically valid tool will provide important insights into the impact of a child’s communication impairment on their functioning and participation at home, at school, and in the community (see [44]).

**Reviewer:**
A discussion of how this tool would be useful for cross-linguistic understanding of language development and disorders would provide more context for this protocol.

It would be useful to discuss a bit more the pros and cons of greater standardization of administration. Should administrators be asked to provide a minimum as well as a maximum of prompts? Would that possibly violate some cultural norms? How could that issue be explored further in future studies?

**Author response:** Thank you for this suggestion. The final two pages of the manuscript have been restructured. The following text has been added to the conclusion section of the manuscript:

Despite our promising results, we acknowledge that using a standardized protocol with scripted follow-up prompts may not be readily achievable across all cultures. In an attempt to avoid ‘leading’ the child to create a personal story the examiner takes a fairly passive role when administering the Global TALES protocol. However, in some cultures children may expect stories to be jointly constructed, for example, and further research is needed to evaluate ways of engaging children in story telling in a culturally responsive, yet standardized way to allow for cross-cultural comparisons. Future research should also investigate the effects of specific prompts and/or conversation partners on children’s ability to provide a coherent personal narrative across different cultures, providing much-needed information to enhance cross-linguistic and cross-cultural understanding of language development and disorders. Findings of such research may indicate that, in some cultures, it may not be appropriate for children to discuss feelings and/or talk about certain topics with unfamiliar adults. Regardless, using a standard protocol to collect normative data across a range of languages and cultures could allow researchers to learn more about this universal discourse genre, its development with age or year of schooling, and how culture may impact performance..

6. PLOS authors have the option to publish the peer review history of their article ([what does this mean?](https://protect-au.mimecast.com/s/jzmjCzvOVBHM5vRDAhMQgil?domain=journals.plos.org)). If published, this will include your full peer review and any attached files.


If you choose “no”, your identity will remain anonymous but your review may still be made public.


**Do you want your identity to be public for this peer review?** For information about this choice, including consent withdrawal, please see our [Privacy Policy](https://protect-au.mimecast.com/s/sNyYCANZjQiNkM90ztBEMkB?domain=plos.org).

Reviewer #1: Yes: Allyssa Mccabe

Reviewer #2: No

**Authors’ response**: we will *opt in* to publish the peer review history.


While revising your submission, please upload your figure files to the Preflight Analysis and Conversion Engine (PACE) digital diagnostic tool, [https://pacev2.apexcovantage.com/](https://protect-au.mimecast.com/s/vXM5CBNZl8i7Y3VqOhQdeAp?domain=pacev2.apexcovantage.com). PACE helps ensure that figures meet PLOS requirements. To use PACE, you must first register as a user. Registration is free. Then, login and navigate to the UPLOAD tab, where you will find detailed instructions on how to use the tool. If you encounter any issues or have any questions when using PACE, please email PLOS at [figures@plos.org](mailto:figures@plos.org). Please note that Supporting Information files do not need this step.

**Author response:**

We have checked the figures using PACE and have inserted them into the body of the MS.
